# Role of Hypoxia-Inducible Factor–1α in Regulating Muscle Degeneration After Rotator Cuff Tears

**DOI:** 10.1177/03635465261469689

**Published:** 2026-08-12

**Authors:** He Zhang, Austin Lee, Mengyao Liu, Agustin Diaz, Yizhao Zhang, Hubert T. Kim, Brian T. Feeley, Xuhui Liu

**Affiliations:** * Department of Physical Education, Central South University, Changsha, China; † Department of Veterans Affairs, San Francisco Veterans Affairs Health Care System, San Francisco, California, USA; ‡ School of Medicine, Case Western Reserve University, Cleveland, Ohio, USA; § Department of Orthopaedic Surgery, University of California, San Francisco, San Francisco, California, USA; Investigation performed at the San Francisco Veterans Affairs Health Care System, San Francisco, California, USA

**Keywords:** HIF-1α, muscle regeneration, fatty infiltration, fibroadipogenic progenitor cell, satellite cell, myocyte

## Abstract

**Background:**

Secondary muscle degeneration after a rotator cuff tear (RCT) critically affects clinical outcomes. Vascular compromise after a tendon injury creates a complex microenvironment that may be associated with the degeneration of rotator cuff muscle. The role of hypoxia-inducible factor–1α (HIF-1α), a master regulator of cellular stress responses to hypoxia, in modulating muscle abnormalities after an RCT remains undefined.

**Purpose:**

To define the role of HIF-1α in stem cell differentiation and muscle degeneration after an RCT in a murine model.

**Study Design:**

Controlled laboratory study.

**Methods:**

A supraspinatus tendon transection and suprascapular nerve transection (TTDN) model was established in C57BL/6J, platelet-derived growth factor receptor α (PDGFRα)–green fluorescent protein (GFP) reporter, and inducible cell-specific HIF-1α knockout mice. Vascularity and HIF-1α colocalization with fibroadipogenic progenitor (FAP) cells and satellite cells were analyzed. Fibrosis, fatty infiltration, and myofiber cross-sectional area were assessed. In vitro, HIF-1α was modulated in isolated FAP cells via CRISPR-Cas9 or prolyl hydroxylase domain inhibitors to evaluate FAP cell differentiation.

**Results:**

TTDN induced significant capillary density reduction (CD31^+^) at 1, 2, and 6 weeks after an injury. Global HIF-1α expression decreased after TTDN compared to the sham side (1 week: 0.78 ± 0.22 vs 1.40 ± 0.42, respectively [*P* = .019]; 2 weeks: 0.74 ± 0.51 vs 1.70 ± 0.48, respectively [*P* = .015]). The percentage of PDGFRα^+^ FAP cells increased at 6 weeks after TTDN compared to the sham side (15.69% ± 1.90% vs 12.76% ± 0.78%, respectively; *P* = .013). The percentage of HIF-1α^+^ FAP cells relative to total PDGFRα^+^ cells significantly decreased in the late stage (6 weeks) of an RCT compared to the sham side (2.78% ± 0.90% vs 7.38% ± 2.29%, respectively; *P* = .003). Knocking out HIF-1α in FAP cells in vivo resulted in increased fibrosis (Cre^+^: 4.43% ± 2.16% vs Cre^–^: 1.72% ± 0.39%; *P* = .047), decreased fatty infiltration (Cre^+^: 0.62% ± 0.42% vs Cre^–^: 1.55% ± 0.45%; *P* = .016), and reduced cross-sectional area (Cre^+^: 664.71 ± 354.45 vs Cre^–^: 1195.81 ± 338.66; *P* = .041). Neither satellite cell–specific nor myocyte-specific HIF-1α deletion resulted in significant phenotypic changes. The downregulation of HIF-1α led to a decrease in uncoupling protein 1 expression and an increase in α–smooth muscle actin expression in FAP cells.

**Conclusion:**

Although vascularity was reduced after TTDN, pronounced global tissue hypoxia was not directly evidenced. Decreased global HIF-1α expression may reflect denervation-induced reductions in metabolic demand. HIF-1α emerges as a key player in FAP cell differentiation within the injury microenvironment, promoting brown adipose tissue differentiation and inhibiting fibrogenesis.

**Clinical Relevance:**

Targeting HIF-1α in FAP cells offers a novel therapeutic strategy to mitigate secondary muscle atrophy and fibrosis after an RCT.

Rotator cuff tears (RCTs) are one of the most prevalent and debilitating joint injuries in middle-aged and older adults.^16,29^ Rotator cuff muscle atrophy and fatty infiltration are proven as independent predictors responsible for the failure of attempted surgical tendon repair that is associated with poor functional outcomes.^3^ However, the detailed mechanisms of rotator cuff muscle degeneration after tendon tears are not fully understood. Understanding the cellular mechanisms of muscle degeneration after an RCT may lead to new therapies for treating muscle degeneration in this setting and provide avenues for treatment in a broader spectrum of muscle injuries.

Oxygen is essential for mammalian cell metabolism and physiological function.^7^ Global or focal reduction of oxygenation can cause muscle atrophy.^22^ Hypoxia-inducible factor (HIF) comprises a family of transcription factors that serve as master regulators of cellular adaptation to stress. HIF consists of an oxygen-dependent α subunit, which is destabilized in normoxia, and a constitutively expressed β subunit.^41^ Overall, 3 HIF-α subunits (HIF-1α, HIF-2α, and HIF-3α) have been described, among which HIF-1α is the best understood and considered the primary mediator of transcriptional responses to both hypoxic and nonhypoxic stress signals.^4,37^ A previous study found that the activation of HIF-1α in murine skeletal myoblasts (under hypoxia in 1% O_2_) leads to an increase of MyoD and promotes myogenesis in vitro.^8^

Fatty infiltration and fibrosis are the major abnormalities of rotator cuff muscle degeneration after tendon tears. HIF-1α also has an important role in regulating fatty infiltration after an injury. It was reported that an RCT induces HIF-1α activation, which in turn activates the transcription of FABP4 (fatty acid–binding protein 4), leading to fat accumulation in injured muscle.^20^ The sustained pharmacological stabilization of HIF-1α induces the expression of collagen and fibronectin, along with the increased expression of profibrotic CTGF/CCN2 (connective tissue growth factor), implying a potential link between chronic hypoxia responses and muscle fibrosis.^38^ Prior studies have identified fibroadipogenic progenitor (FAP) cells as the primary cellular source of adipose and fibrotic tissue in rotator cuff muscle.^25^ Nevertheless, the precise role of HIF-1α in FAP cell–mediated adipogenesis and fibrogenesis remains incompletely elucidated.

The aim of this study was to characterize the expression of HIF-1α in rotator cuff muscle using a mouse model of RCTs. We intended to investigate the role of HIF-1α in FAP cell differentiation as well as fatty infiltration and fibrosis in rotator cuff muscle. Furthermore, we sought to explore the role of HIF-1α in various cell populations within rotator cuff muscle, including myocytes, FAP cells, and satellite cells (SCs), throughout the progression of muscle fibrosis and fatty infiltration after an RCT, employing cell-specific Cre-lox HIF-1α knockout mice.

## Methods

### Animals

In this study, 3-month-old male C57BL/6J mice (stock No. 000664; The Jackson Laboratory) were used. Additionally, platelet-derived growth factor receptor α (PDGFRα)–green fluorescent protein (GFP) reporter mice (stock No. 007669; The Jackson Laboratory), which express the H2B-EGFP fusion gene from the endogenous PDGFRα locus, were employed to trace PDGFRα^+^ FAP cells during rotator cuff muscle degeneration after tendon tears. Inducible cell-specific HIF-1α knockout mice were generated by crossing HIF-1α lox mice (strain No. 007561; The Jackson Laboratory) with tamoxifen-inducible PDGFRα Cre-ERT (for FAP cells; strain No. 018280 [The Jackson Laboratory]), Pax7 Cre-ERT (for SCs; strain No. 017763 [The Jackson Laboratory]), or ACTA1 Cre-ERT (for mature myocytes; strain No. 025750 [The Jackson Laboratory]). Tamoxifen was administered to all Cre-inducible mouse strains via an intraperitoneal injection at a dose of 80 mg/kg body weight for 5 consecutive days.^6^ Target cell populations were isolated by fluorescence-activated cell sorting (FACS), and genomic DNA was extracted and analyzed by PCR using primers flanking the loxP sites to confirm deletion of the floxed *Hif1a* exon. Littermate Cre^–^/HIF-1α^lox/lox^ mice in each strain served as controls. A detailed summary of the experimental groups can be found in Table 1.

**Table 1:** Experimental Groups*^*a*^*

Type of Mice	Sex	Sample Size	Harvest Time Point, wk	Assays Performed
C57BL/6J	Male	15 (5 per time point)	1, 2, and 6	Immunofluorescence, Western blotting, FACS
PDGFRα-GFP reporter	Male	15 (5 per time point)	1, 2, and 6	Immunofluorescence
Cre^+^/HIF-1α^lox/lox^ (PDGFRα Cre/Pax7 Cre/ACTA1 Cre)	Male	15 (5 per strain)	6	Histology (Masson’s trichrome/Oil Red O), immunofluorescence
Cre^–^/HIF-1α^lox/lox^ (PDGFRα Cre/Pax7 Cre/ACTA1 Cre)	Male	15 (5 per strain)	6	Histology (Masson’s trichrome/Oil Red O), immunofluorescence

a FACS, fluorescence-activated cell sorting.

All the mice were housed in a neutral temperature environment on a 12-hour/12-hour light/dark cycle and provided with standard laboratory food and water. All animal procedures were approved by an Institutional Animal Care and Use Committee (No. 24-006).

### Surgical Procedures

Mice underwent unilateral supraspinatus (SS) tendon transection and suprascapular nerve transection (TTDN) as previously described.^23^ Sham surgery on the contralateral side served as a control, involving a skin incision and tendon/nerve exposure, followed by immediate closure. C57BL/6J mice and PDGFRα-GFP reporter mice were humanely sacrificed at 1, 2, and 6 weeks after surgery. Bilateral SS muscles were harvested, snap-frozen, and cryosectioned for histological analysis. A total of 5 mice per group per time point was used. Cell-specific HIF-1α knockout mice (Cre^+^/HIF-1α^lox/lox^) and their littermate Cre^–^/HIF-1α^lox/lox^ mice were sacrificed at 6 weeks after unilateral TTDN (n = 5 per group). Subsequently, SS muscles were harvested for histological analysis. All the surgical procedures were conducted under general anesthesia using 1% to 5% isoflurane in oxygen.

### Muscle Harvest and Histology

Muscle specimens were flash-frozen by immersion in liquid nitrogen–cooled isopentane and sectioned in the transverse plane (perpendicular to the longitudinal axis of the muscle fibers) with a 7-µm thickness. For immunofluorescence staining, slides were fixed with 4% paraformaldehyde for 10 minutes, washed in 1× phosphate-buffered saline (PBS) with 0.1% Triton X-100, blocked in 5% bovine serum albumin for 1 hour, and incubated with primary antibodies at 4°C overnight. Slides were then washed in PBS and incubated with secondary antibodies (1:200) for 1 hour at room temperature. Tissue sections were stained with DAPI (4′,6-diamidino-2-phenylindole) and then mounted with Fluoromount-G. Vascularity was determined as an area positive for CD31 staining divided by total muscle fibers.^33^ Laminin was stained to assess muscle cross-sectional area (CSA). HIF-1α immunostaning was combined with Pax7 staining or PDGFRα-GFP reporter detection to assess HIF-1α localization in Pax7^+^ SCs and PDGFRα^+^ FAPs, respectively. Masson's trichrome (American MasterTech Scientific) and Oil Red O (Sigma-Aldrich) stains were used to assess fibrosis (fibrotic area/total muscle area) and fatty infiltration (at area/total muscle area) in SS muscles. Quantification was conducted using ImageJ. The slides were independently reviewed by 2 blinded reviewers, and at least 5 randomly chosen locations in the muscle belly for each sample were assessed.

### FAP Cell Isolation and Culture

Primary FAP cells were isolated from SS muscles of C57BL/6J mice by FACS.^19^ In brief, muscles were minced into small pieces with sterile scissors in a cell culture hood and incubated with 0.2% collagenase type II for 90 minutes in a 37°C sterile water bath. Washing buffer (F-10, 10% horse serum, and 1× HEPES) was added into the mixture and centrifuged at 1500 rpm for 5 minutes at room temperature. The remnant was rinsed with washing buffer and centrifuged at 1500 rpm for 5 minutes. D/2 solution (0.06% collagenase type II and 0.15% dispase with washing buffer) was then added and incubated for 30 minutes at 37°C. The solution was then passed through a 70-μm cell strainer (VWR International), followed by a 40-μm cell strainer (VWR International). The filtered cells were washed with 40 mL of FACS buffer (2.5% fetal bovine serum, 20 mM EDTA, and 1× PBS) and spun at 1500 rpm for 5 minutes. The supernatant was discarded, and the cell pellets were resuspended with 500 μL of FACS buffer. The cells were incubated with anti–CD31-FITC (fluorescein isothiocyanate) (clone 390; BD Biosciences), anti–CD45-FITC (clone WM59; BD Biosciences), anti–integrin α7–allophycocyanin (clone 334908; R&D Systems), and phycoerythrin-Cy7–Sca-1 (clone E13-161.7; BD Biosciences) for 30 minutes before being sorted with FACSAria II (BD Biosciences). FAP cells were collected as the CD45^−^/CD31^−^/integrin α7^−^/Sca-1^+^/CD140a^+^ cell population.^18,24^ After sorting, FAP cells were seeded onto 1% Matrigel (Corning)–precoated cell plates and cultured in standard F-10 medium supplemented with 10% fetal bovine serum, 100 ng/mL of basic fibroblast growth factor, and 1% antibiotic-antimycotic solution under normoxic conditions.

### Cell Transfection

CRISPR-Cas9 technology was employed to knock down HIF-1α in FAP cells using the Gene Knockout Kit (Version 2; Synthego) and single guide RNA (sgRNA) for HIF-1α. Lipofectamine RNAiMAX Reagent (Thermo Fisher Scientific) was utilized for all transfections following the manufacturer's instructions. Treated FAP cells were cultured for an additional 10 days to allow FAP cell differentiation before being harvested for mRNA and protein extraction. The efficiency of HIF knockdown was assessed by Western blot analysis for HIF-1α. FAP cell differentiation was evaluated with real-time PCR and immunostaining of fibrogenic, white adipogenic, and beige adipogenic markers.

### Prolyl Hydroxylase Domain (PHD) Inhibitor Treatment

Under normoxic conditions, HIF-1α proteins are constitutively hydroxylated at conserved proline residues by PHD enzymes, targeting them for Von Hippel-Lindau syndrome–mediated ubiquitination and proteasomal degradation.^31^ To pharmacologically stabilize HIF-1α proteins and activate downstream HIF-1α transcriptional programs, cells were treated with MK-8617 (50 nM; MedKoo Biosciences),^21^ a potent pan-inhibitor of PHD1, PHD2, and PHD3 that prevents prolyl hydroxylation–dependent HIF-1α degradation, thereby facilitating HIF-1α nuclear translocation and hormone response element–driven target gene transcription.^30^ Cells in control groups were treated with 10% dimethyl sulfoxide (DMSO) in the same amount.

### Western Blotting

Protein extraction and Western blotting were conducted as previously reported.^46^ Proteins extracted from SS muscles were obtained using T-PER Tissue Protein Extraction Reagent (No. 78510; Thermo Fisher Scientific), while proteins from FAP cells were extracted using M-PER Mammalian Protein Extraction Reagent (No. 78505; Thermo Fisher Scientific). These protein extraction solutions were supplemented with protease inhibitor cocktail (04693116001; Roche) and phosphatase inhibitor cocktail (A32957; Thermo Fisher Scientific). Protein concentrations were measured using the Pierce BCA Protein Assay Kit (No. 23225; Thermo Fisher Scientific). Subsequently, the samples were boiled with loading buffer at 98°C for 5 minutes, separated using 4% to 12% NuPAGE Bis-Tris Gel (Thermo Fisher Scientific), and transferred onto polyvinylidene difluoride membranes (88518; Thermo Fisher Scientific). The membranes were then blocked in tris-buffered saline with 0.1% Tween 20 containing 5% skim milk at room temperature for 30 minutes, followed by overnight incubation with primary antibodies at 4°C. After washing the membranes with tris-buffered saline with 0.1% Tween 20 three times for 5 minutes each, they were incubated with secondary antibodies for 1 hour at room temperature. Immunoreactive bands were visualized by the Odyssey system (LI-COR Biosciences). GAPDH (glyceraldehyde 3-phosphate dehydrogenase) was used as the loading control, and quantification was conducted using ImageJ.

### Real-Time PCR

RNA extraction and real-time PCR were conducted as previously reported.^45^ Primer sequences of the genes tested are summarized in Table 2. To evaluate phenotypic changes, we assessed the expression of HIF-1α, beige adipogenic markers (uncoupling protein 1 [UCP1] and PRDM16), white adipogenic markers (adiponectin, PPARγ, and CEBPα), and fibrogenic markers (collagen Iα, collagen IIIα, and α–smooth muscle actin [α-SMA]). The expression level of each gene was normalized to that of the housekeeping gene of 18S ribosomal RNA. The fold difference relative to controls was calculated using the double delta cycle threshold method. Three biological replicates were included in each experiment, with each sample analyzed in technical triplicate by RT-qPCR.

**Table 2:** Gene Primer Sequences

Gene	Forward (5′-3′)	Reverse (5′-3′)
HIF-1α	CTATGGAGGCCAGAAGAGGGTAT	CCCACATCAGGTGGCTCATAA
UCP1	AGGCTTCCAGTACCATTAGGT	CTGAGTGAGGCAAAGCTGATTT
PRDM16	TATGGAGCTAGGCAGGGACA	TCCATACATCAGGGAGCAGA
Adiponectin	CCCAAGGGAACTTGTGCAGGTTGGATG	GTTGGTATCATGGTAGAGAAGAAAGCC
PPARγ	GCATGGTGCCTTCGCTGA	TGGCATCTCTGTGTCAACCATG
CEBPα	AACCTCATCCGCCACCTG	GTAGACAACAGCCGCATCC
Collagen Iα	CAGCCGCTTCACCTACAGC	TTTTGTATTCAATCACTGTCTTGCC
Collagen IIIα	CAGGACCTAAGGGCGAAGATG	TCCGGGCATACCCCGTATC
α-SMA	GGACGTACAACTGGTATTGTGC	TCGGCAGTAGTCACGAAGGA
18S	CTCTGTTCCGCCTAGTCCTG	AATGAGCCATTCGCAGTTTC

### Antibodies

Primary antibodies and concentrations used for Western blotting and immunofluorescence in this study were as follows: GAPDH (1:2000; PAB0907 [Abnova]), β-actin (1:2000; ab8227 [Abcam]), rabbit anti-mouse laminin (1:200; PA1-16730 [Thermo Fisher Scientific]), rat anti-mouse laminin (1:400; ab11576 [Abcam]), rabbit anti-mouse HIF-1α (1:1000 for Western blotting/1:200 for immunofluorescence; ab2185 [Abcam]), mouse anti-mouse MF20 (1:50; Developmental Studies Hybridoma Bank), rat anti-mouse CD31 (1:100; 102502 [BioLegend]), rabbit anti-mouse α-SMA (1:200; SAB5500002 [Sigma-Aldrich]), and goat anti-mouse perilipin (1:200; SAB2500775 [Sigma-Aldrich]).

Secondary antibodies used in this study were as follows: donkey anti-rabbit IRDye 800CW (1:1000; 925-32213 [LI-COR Biosciences]), donkey anti-rabbit IgG H&L Alexa Fluor 647 (1:200; A31573 [Thermo Fisher Scientific]), donkey anti-rabbit IgG H&L Alexa Fluor 488 (1:200; ab150073 [Abcam]), donkey anti-mouse IgG H&L Alexa Fluor 594 (1:200; ab150108 [Abcam]), donkey anti-mouse IgG H&L Alexa Fluor 647 (1:200; ab150107 [Abcam]), goat anti-rat IgG H&L Alexa Fluor 594 (1:200; ab150160 [Abcam]), goat anti-rat IgG H&L Alexa Fluor 488 (1:200; ab150157 [Abcam]), and goat anti-rat IgG H&L Alexa Fluor 647 (1:200; ab150159 [Abcam]).

### Statistical Analysis

Sample sizes were determined based on our previous studies with the same TTDN mouse model^6,46^ and preliminary experimental data to achieve adequate statistical power (power ≥ 0.80; α = 0.05). The homogeneity of variance was assessed using the Levene test. For comparisons between 2 groups, the unpaired Student's *t*-test was applied when data satisfied assumptions of normality and equal variance. The Welch's *t*-test was used when equal variance could not be assumed. All data were analyzed with Prism (Version 8; GraphPad Software) and shown as the mean ± standard deviation. Statistical significance was determined when *P* < .05.

## Results

### Vascularity in SS Muscles After Injury

**Figure 1.**
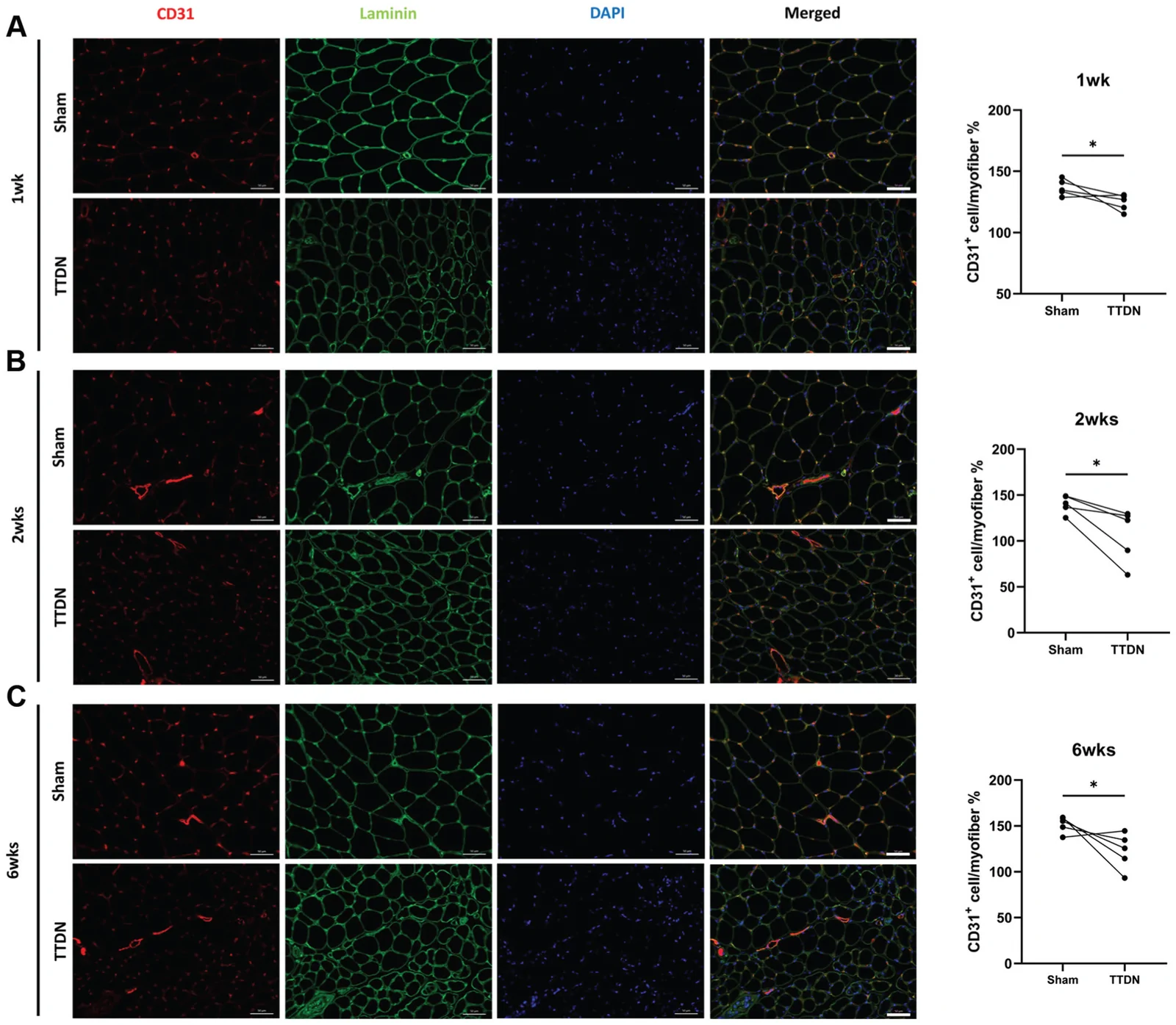
Representative images of immunofluorescence for vascularity in SS muscles at (A) 1 week, (B) 2 weeks, and (C) 6 weeks after TTDN (n = 5 per time point). Quantitative results of vascularity via immunofluorescence are presented as mean ± SD. The sham side was used as the control. Blood vessels were stained with CD31 (red), while the myofiber membrane was stained with laminin (green). DAPI was used to visualize nuclei (blue). Scale bar: 50 μm. *P < .05 by the Student's *t*-test.

Immunofluorescence staining with CD31 revealed a significant decrease in vascularity after the RCT that initially progressed over time. The percentage of CD31^+^ endothelial cells/myofibers significantly reduced after TTDN compared to the sham side at 1 week (126.60% ± 6.63% vs 136.59% ± 6.50%, respectively; *P* = .02) (Figure 1A), 2 weeks (106.50% ± 29.12% vs 140.98% ± 9.80%, respectively; *P* = .04) (Figure 1B), and 6 weeks (122.48% ± 19.75% vs 151.84% ± 8.91%, respectively; *P* = .016) (Figure 1C).

### HIF-1α Expression in SS Muscles After Injury

**Figure 2.**
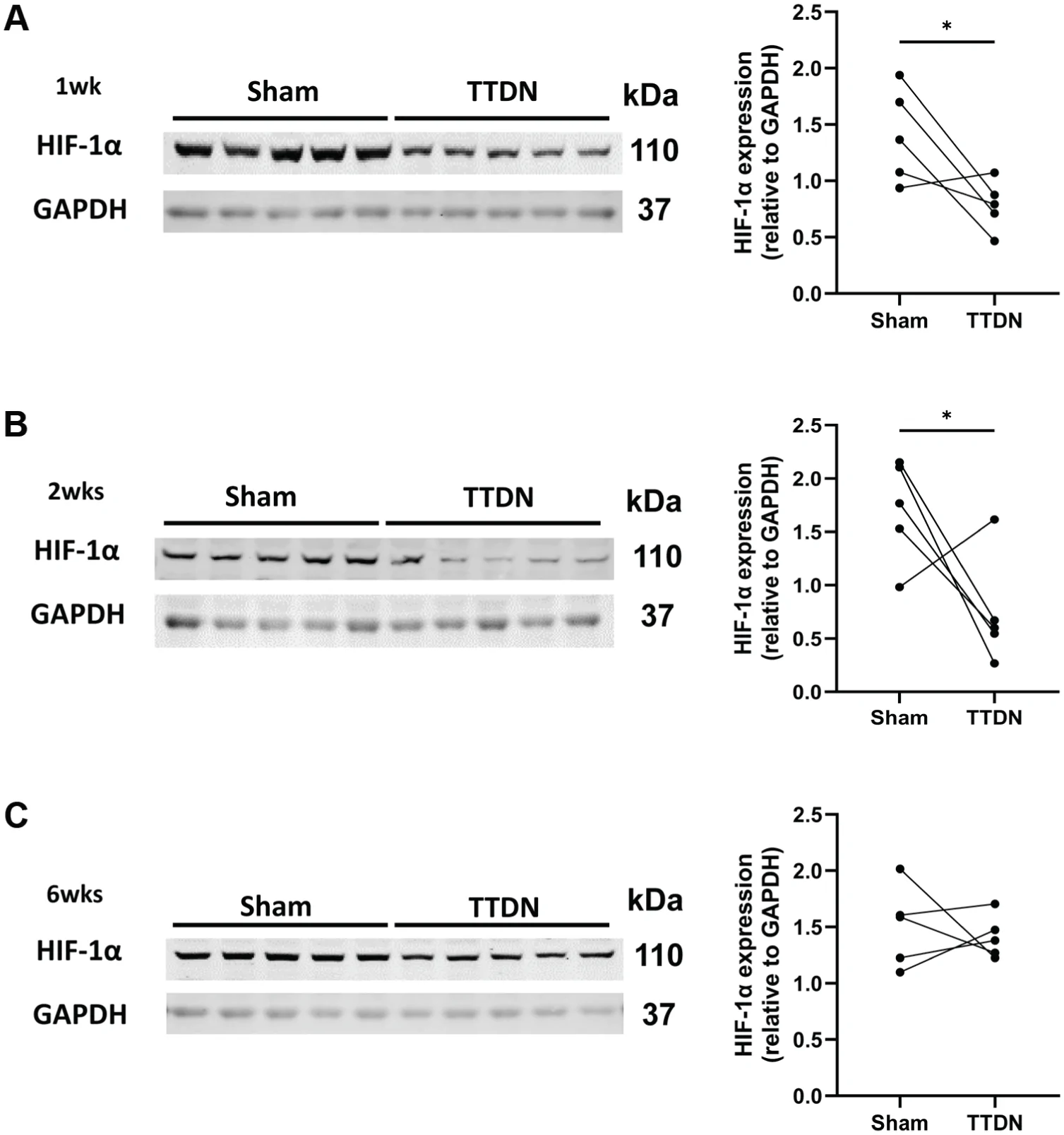
Protein levels of HIF-1α at (A) 1 week, (B) 2 weeks, and (C) 6 weeks after TTDN (n = 5 per time point). All data are presented with the relative value normalized to GAPDH. *P < .05 by the Student's *t*-test.

We then measured the protein expression of HIF-1α at different time points after the RCT. Western blot analysis revealed a significant reduction in protein expression at both 1 week (0.78 ± 0.22 vs 1.40 ± 0.42, respectively; *P* = .019) and 2 weeks (0.74 ± 0.51 vs 1.70 ± 0.48, respectively; *P* = .015) after TTDN compared to the sham side (Figure 2, A and B). However, no significant change was observed at the 6-week time point (1.41 ± 0.19 vs 1.50 ± 0.36, respectively; *P* = .615) (Figure 2C).

### HIF-1α Expression in SCs After Injury

**Figure 3.**
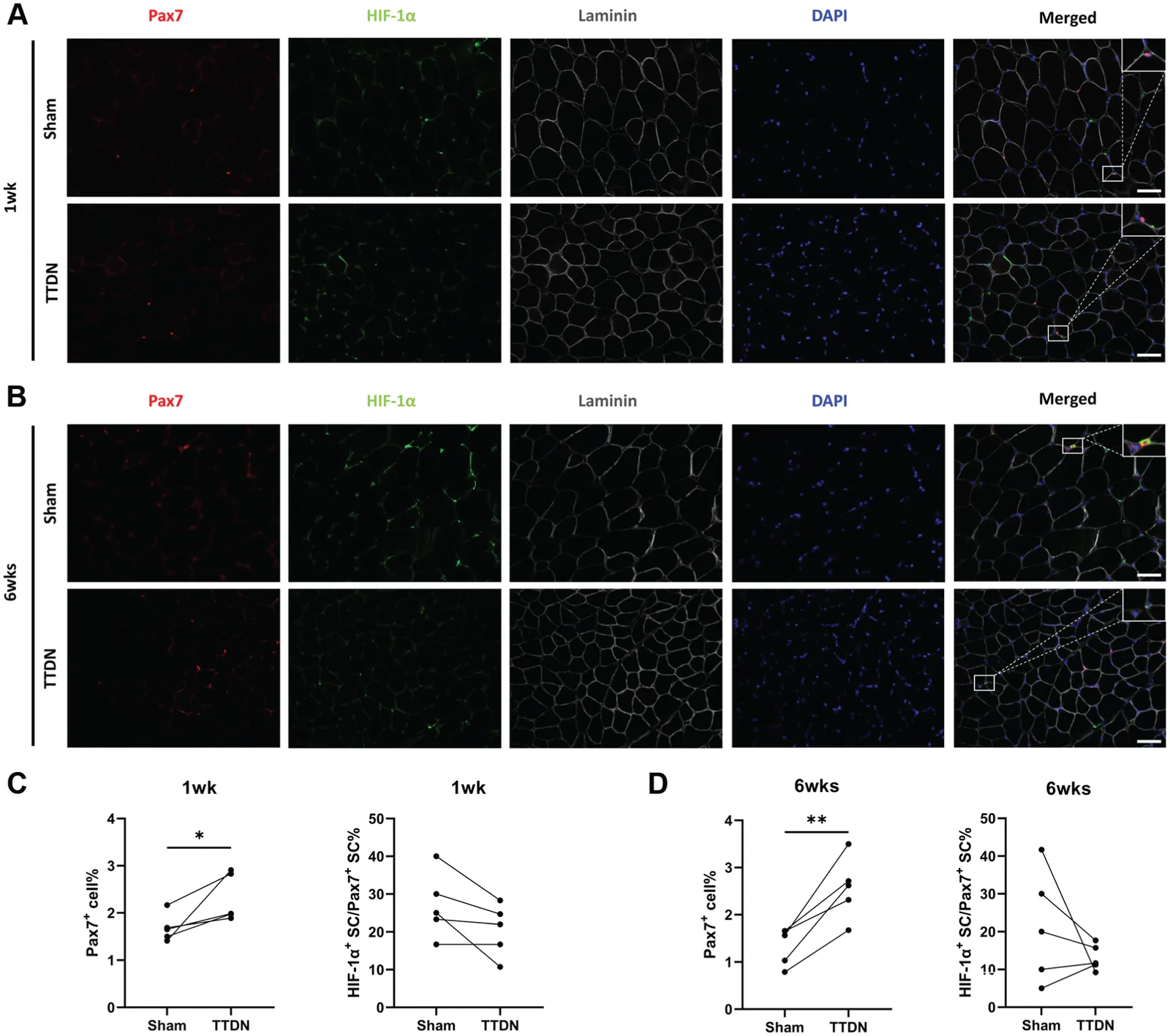
Representative images of immunostaining showing the overlap between HIF-1α^+^ SCs at (A) 1 week and (B) 6 weeks after TTDN (n = 5 per time point). Quantification of Pax7^+^ SCs as a percentage of total cells and HIF-1α^+^ SCs as a percentage of Pax7^+^ cells at (C) 1 week and (D) 6 weeks. SCs were stained with Pax7 in red, HIF-1α was stained in green, and the myofiber membrane was stained with laminin in gray. DAPI was used to visualize nuclei in blue. Scale bar: 50 μm. *P < .05 and **P < .01 by the Student's *t*-test.

**Figure 4.**
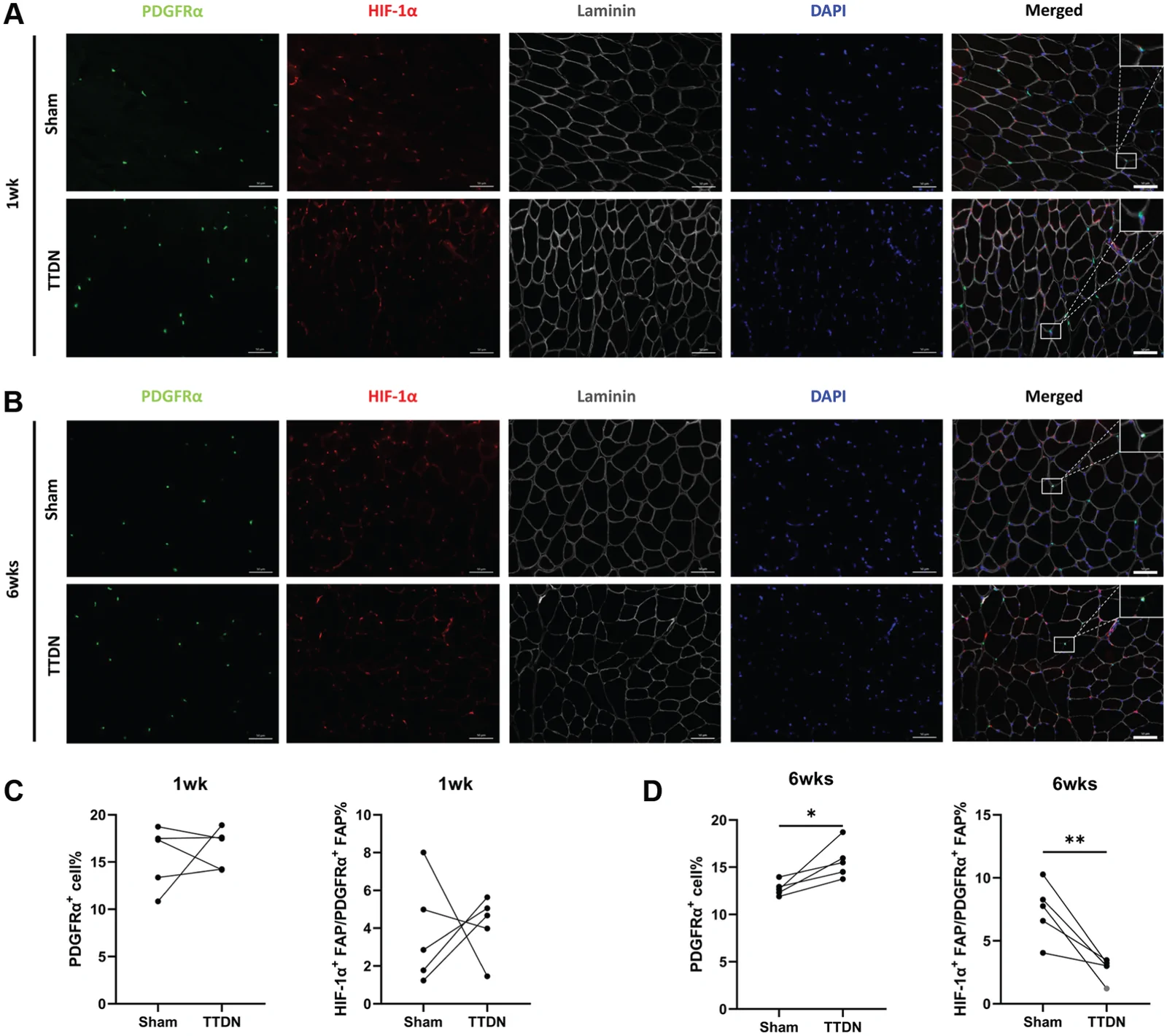
Representative images of immunostaining showing the overlap between HIF-1α^+^ FAP cells at (A) 1 week and (B) 6 weeks after TTDN (n = 5 per time point). Quantification of PDGFRα^+^ FAP cells as a percentage of total cells and HIF-1α^+^ FAP cells as a percentage of PDGFRα^+^ cells at (C) 1 week and (D) 6 weeks. FAP cells were stained with PDGFRα in green, HIF-1α was stained in red, and the myofiber membrane was stained with laminin in gray. DAPI was used to visualize nuclei in blue. Scale bar: 50 μm. *P < .05 and **P < .01 by the Student's *t*-test.

Our next objective was to characterize HIF-1α expression in 2 vital stem cell populations in rotator cuff muscle after the injury: SCs and FAP cells. Immunostaining for the SC marker, Pax7, and FAP cell marker, PDGFRα, was performed along with HIF-1α staining at 1 and 6 weeks after TTDN (Figures 3 and 4). After TTDN, the percentage of Pax7^+^ SCs relative to total cells significantly increased compared to the sham side at both 1 week (2.32% ± 0.50% vs 1.68% ± 0.29%, respectively; *P* = .041) (Figure 3, A and C) and 6 weeks (2.56% ± 0.66% vs 1.34% ± 0.40%, respectively; *P* = .008) (Figure 3, B and D), implying the activation of SCs for muscle repair. No noticeable change was observed in the proportion of HIF-1α^+^/Pax7^+^ double-positive cells among total Pax7^+^ SCs between injured muscles and their contralateral counterparts at both time points (*P* > .05) (Figure 3, C and D).

### HIF-1α Expression in FAP Cells After Injury

The percentage of PDGFRα^+^ FAP cells relative to total cells increased at 6 weeks after TTDN compared to the sham side (15.69% ± 1.90% vs 12.76% ± 0.78%, respectively; *P* = .013) (Figure 4, B and D). No significant change was observed in the percentage of HIF-1α^+^ FAP cells relative to total PDGFRα^+^ cells at 1 week (4.16% ± 1.63% vs 3.48% ± 2.25%, respectively; *P* = .792) (Figure 4, A and C). However, it significantly decreased at 6 weeks (2.78% ± 0.90% vs 7.38% ± 2.29%, respectively; *P* = .003) (Figure 4, B and D).

### Morphological Changes in SS Muscles in Cell-Specific HIF-1α Knockout Mice After Injury

**Figure 5.**
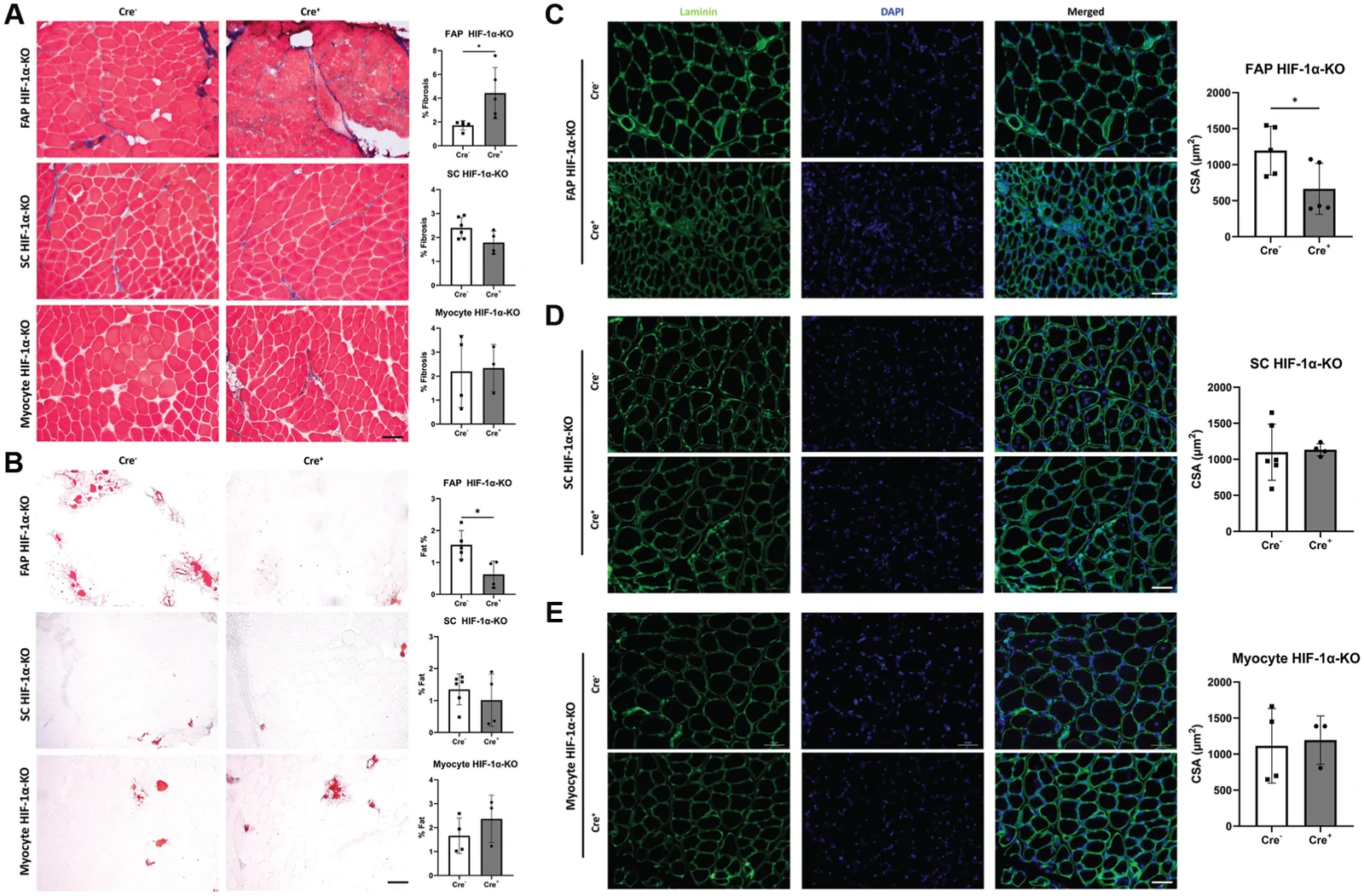
Representative images of (A) Masson's trichrome staining and (B) Oil Red O staining as well as quantification of fibrosis/fatty infiltration in FAP cell, SC, and myocyte Cre^+^/HIF-1α^lox/lox^ knockout mice along with their littermate Cre^–^ control mice at 6 weeks after an injury. Representative images of immunostaining and quantification of CSA in (C) FAP cell, (D) SC, and (E) myocyte Cre^+^/HIF-1α^lox/lox^ knockout mice and their littermate Cre^–^ control mice at 6 weeks after an injury. The myofiber membrane was stained with laminin in gray. DAPI was used to visualize nuclei in blue. Scale bar: 50 μm. Because of animal mortality during the husbandry and experimental procedures, the final sample sizes included 4 Pax7 Cre^+^/HIF-1α^lox/lox^ mice, 4 ACTA1 Cre^–^/HIF-1α^lox/lox^ mice, and 3 ACTA1 Cre^+^/HIF-1α^lox/lox^ mice. The sample size for all other groups was maintained at 5 mice each. *P < .05 by the Student's *t*-test for data with equal variances and Welch's *t*-test for data with unequal variances.

We next sought to define the role of HIF-1α in different cell populations in rotator cuff muscle—myocytes, FAP cells, and SCs—during muscle degeneration after an RCT, utilizing cell-specific HIF-1α Cre-lox knockout mice. Masson's trichrome staining revealed a significant increase in fibrosis exclusively in PDGFRα Cre^+^/HIF-1α^lox/lox^ mice compared to the littermate Cre^–^ control mice (4.43% ± 2.16% vs 1.72% ± 0.39%, respectively; *P* = .047) (Figure 5A). Additionally, fatty infiltration showed a significant decrease in PDGFRα Cre^+^/HIF-1α^lox/lox^ mice compared to the littermate Cre^–^ control mice (0.62% ± 0.42% vs 1.55% ± 0.45%, respectively; *P* = .016) (Figure 5B). No significant differences in fibrosis and fatty infiltration were observed in the Pax7 Cre^+^/HIF-1α^lox/lox^ and ACTA1 Cre^+^/HIF-1α^lox/lox^ mice (*P* > .05) (Figure 5, A and B). Immunostaining results revealed a decrease in myofiber size after the injury in PDGFRα Cre^+^/HIF-1α^lox/lox^ mice compared to the littermate Cre^–^ control mice (664.71 ± 354.45 vs 1195.81 ± 338.66, respectively; *P* = .041) (Figure 5C). No significant difference in CSA was observed between the Pax7 Cre^+^/HIF-1α^lox/lox^ and ACTA1 Cre^+^/HIF-1α^lox/lox^ mice compared to their littermate Cre^–^ control mice (*P* > .05) (Figure 5, D and E). Among these 3 strains, a phenotype was only observed in FAP cell–specific HIF-1α knockout mice, suggesting that HIF-1α plays a more important role in FAP cells than SCs and myocytes during rotator cuff muscle degeneration.

### Effect of HIF-1α Regulation on Fibrogenesis and Adipogenesis of FAP Cells

**Figure 6.**
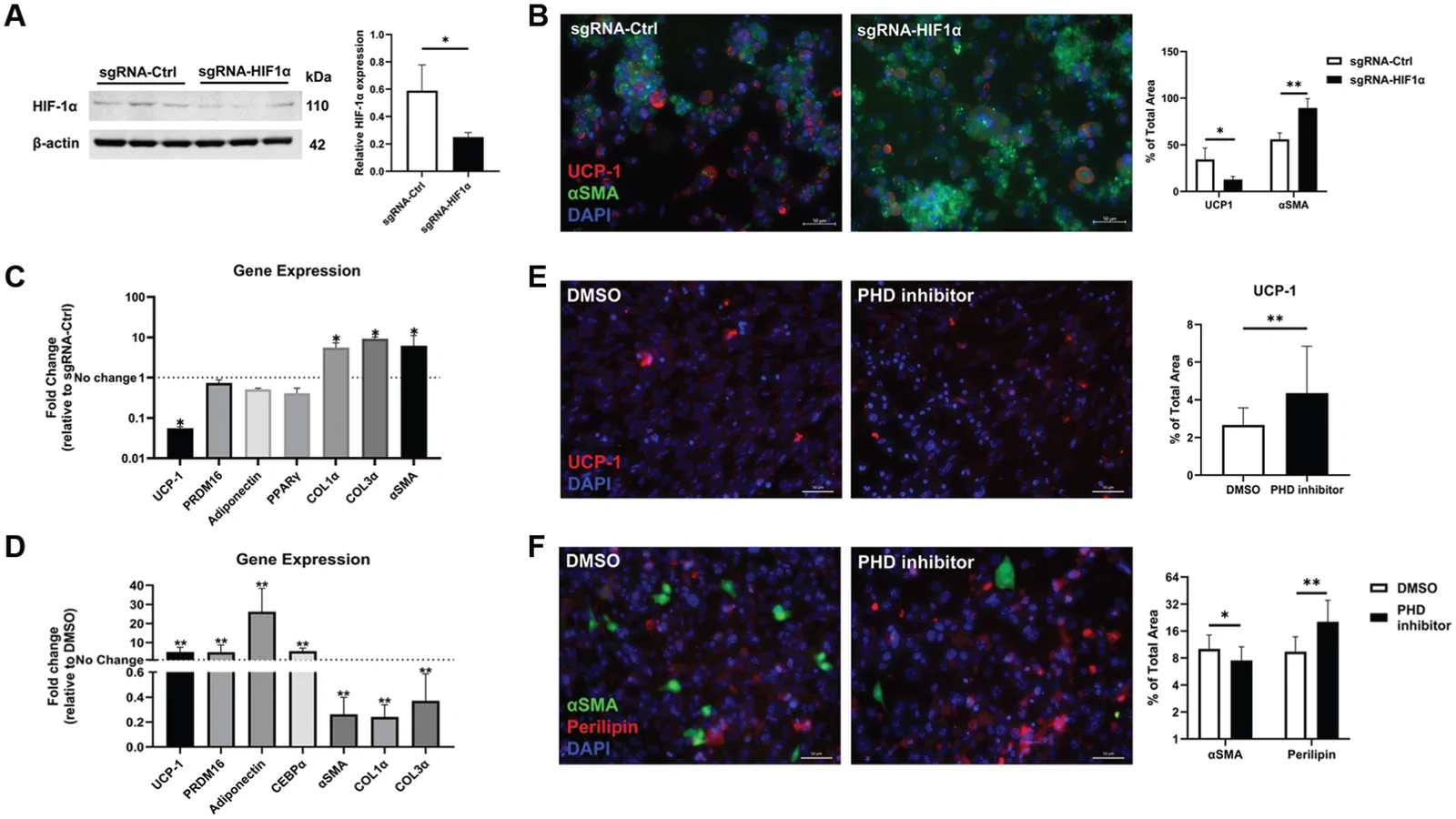
(A) Protein expression of HIF-1α in FAP cells transfected with sgRNA compared to the control group. (B) Representative immunostaining images and quantification for UCP1 and α-SMA in FAP cells transfected with sgRNA. Scale bar: 50 μm. (C) Comparative gene expression profile from sgRNA-HIF-1α–treated FAP cells normalized to FAP cells from the control group. 18S was used as the housekeeping gene. (D) Comparative gene expression profile from PHD inhibitor–treated FAP cells normalized to FAP cells from the DMSO group. 18S was used as the housekeeping gene. Representative immunostaining images for (E) UCP1 and (F) α-SMA and perilipin in FAP cells treated with the PHD inhibitor. *P < .05 and **P < .01 by the Student's *t*-test for data with equal variances and Welch's *t*-test for data with unequal variances.

To further evaluate the function of HIF-1α on FAP cell differentiation, we modulated its expression using a PHD inhibitor and the Gene Knockout Kit with sgRNA for HIF-1α and then assessed the fibrogenesis and adipogenesis of FAP cells in vitro. Western blot results showed that sgRNA transfection significantly reduced the protein expression of HIF-1α compared to the control group (0.25 ± 0.03 vs 0.58 ± 0.19, respectively; *P* = .038) (Figure 6A). Knocking down of HIF-1α significantly decreased UCP1 expression (12.87% ± 3.25% vs 34.3% ± 12.30%, respectively; *P* = .036) but increased α-SMA expression in FAP cells (89.28% ± 10.11% vs 55.90% ± 6.91%, respectively; *P* = .003) (Figure 6B). Consistent with the immunostaining results, Real-time PCR further confirmed a significant increase in the mRNA levels of fibrogenic markers and a significant decrease in those of adipogenic markers in FAP cells after HIF-1α knockdown (Figure 6C). MK-8617 treatment was associated with significantly elevated HIF-1α mRNA levels in FAP cells, confirming the transcriptional presence of HIF-1α in the injury microenvironment. These findings are interpreted as reflecting basal and injury-responsive HIF-1α transcriptional activity rather than a direct transcriptional effect of PHD inhibition (PHD inhibitor: 46.84 ± 17.32 vs DMSO: 1.03 ± 0.31; *P* = .044) (Appendix Figure A1, available in the online version of this article). The pharmacological stabilization of HIF-1α proteins via MK-8617 treatment was associated with the increased mRNA expression of brown/beige adipogenic transcriptional regulators, including UCP1 and PRDM16, as well as broader adipogenic marker genes, alongside the downregulation of fibrogenic markers, including α-SMA, collagen Iα, and collagen IIIα (Figure 6D). Immunostaining also revealed increased UCP1 expression with MK-8617 treatment (PHD inhibitor: 4.35% ± 2.49% vs DMSO: 2.66% ± 0.91%; *P* = .002) (Figure 6E). Additionally, MK-8617 treatment was associated with a significant reduction in α-SMA–positive FAP cells (PHD inhibitor: 7.51% ± 3.12% vs DMSO: 10.11% ± 4.33%; *P* = .031), accompanied by a significant increase in perilipin-positive FAP cells (PHD inhibitor: 20.26% ± 15.11% vs DMSO: 9.39% ± 4.42%; *P* = .005) (Figure 6F).

## Discussion

Secondary muscle degeneration after RCTs involves a complex interplay of cellular and molecular mechanisms within the injury microenvironment. Injury-induced vascular compromise can potentially alter the metabolic microenvironment of affected tissue. However, whether vascular compromise translates to true tissue hypoxia depends on the balance between oxygen supply and cellular oxygen consumption. Previous studies have reported decreased capillary density and activated hypoxia signaling in different models of skeletal muscle damage.^2,39^ In our study, we observed a significant decrease in vascularity following the induction of a clinically relevant rotator cuff tear (Figure 1), indicating vascular compromise that may potentially contribute to altered tissue oxygenation. The transcription factor HIF-1 is believed to be one of the most important regulators of cellular responses to hypoxia, although accumulating evidence has demonstrated that HIF-1 also can be activated through oxygen-independent mechanisms.^1,14,17^ In this study, we found that the expression of HIF-1α proteins in SS muscles significantly decreased in the early stage of the RCT and returned to baseline levels in the late stage (6 weeks) after TTDN. This finding appears counterintuitive, but it is important to note that HIF-1α expression levels do not directly reflect tissue oxygenation status, and the absence of elevated global HIF-1α expression does not indicate the absence of hypoxia or other stress signals. The regulation of HIF-1α expression likely reflects a complex interaction of multiple factors within the injury microenvironment. HIF-1α transcription and protein stability are regulated not only by oxygen availability but also by inflammatory resolution, metabolic adaptation, and other mechanisms.^47^ The temporal downregulation of global HIF-1α may reflect denervation-induced reductions in overall cellular metabolic activity and oxygen consumption, shifts in the cellular composition of muscle, or changes in the inflammatory milieu.

SCs are among the muscle-resident progenitor cells primarily responsible for muscle regeneration after an injury.^9,12^ Under normal conditions, SCs remain in a quiescent state between the sarcolemma and the basal lamina of myofibers, characterized by expression of the transcription factor Pax7.^36^ An injury triggers the activation of SCs, promoting them to re-enter the cell cycle, proliferate, and subsequently differentiate into myoblasts. These myoblasts then undergo extensive proliferation and migration toward the injury site, where they undergo myogenic differentiation to fuse with new or existing myofibers.^35^ Results in this study showed an increase in Pax7^+^ SCs, which is consistent with a previous study,^11^ indicating the proliferation and activation of SCs in rotator cuff muscle after an injury. However, no significant change was observed in HIF-1α expression in SCs.

The role of HIF-mediated signaling in adult SCs is complex. Recent evidence has highlighted HIF-1α as a regulator of myogenesis and SC function.^32^ Double knockout of HIF-1α and HIF-2α in SCs showed delayed regeneration after an injury and self-renewal inhibition of SCs under hypoxic conditions.^44^ Knocking down of HIF-1α by siRNA inhibits myogenesis in C2C12 myoblasts as well.^34^ Conversely, a study reported that HIF-1α deletion in SCs accelerates regeneration and myogenesis in ischemia experiments, suggesting that HIF-1α may negatively regulate regeneration through SC proliferation.^27^ Additionally, an in vitro study in myoblasts showed that the activation of HIF-1α impaired myogenesis under hypoxic conditions.^28^

Therefore, to further understand the role of HIF-1α in SCs and myocytes during muscle regeneration after RCTs, we utilized SC/myocyte HIF-1α knockout mice and measured muscle fiber size, fatty infiltration, and fibrosis after TTDN. The data revealed no significant difference in these indexes in both strains (Figures 5 and 6). These findings suggest that the absence of HIF-1α in SCs and myocytes has minimal impact on muscle morphology after RCTs. While there was an increase in the number of SCs, the observed muscle changes after an RCT predominantly reflect degenerative processes rather than regenerative ones. Despite this increase in the number of SCs, no regenerative myofibers were observed in SS muscles after the RCT. Therefore, the increase in SC after RCT does not translate into complete myogenesis or myofiber regeneration in the SS muscle, potentially because the absence of rotator cuff repair deprives the muscle of the regenerative cues required to initiate or sustain this process. The incomplete myogenesis of SCs results in the limited impact of HIF-1α deficiency, as observed in this study.

Another progenitor cell population that contributes to muscle degeneration after RCTs is FAP cells. After a muscle injury, FAP cells rapidly proliferate and provide prodifferentiation signaling for myogenic precursors.^13,15^ It has been reported that FAP cells are the cellular source of fatty infiltration and fibrosis in rotator cuff muscle after an injury.^25^ However, the relationship between FAP cells and HIF-1α has not been described. Our results demonstrated a significant rise in the number of PDGFRα^+^ FAP cells after an RCT, which is consistent with previous work.^10^ The proportion of HIF-1α–expressing FAP cells (HIF-1α^+^/PDGFRα^+^ FAP cells) was significantly reduced in the late stage of the injury. By utilizing FAP cell–specific HIF-1α knockout mice, we observed significantly increased fibrosis, decreased fatty infiltration, and reduced myofiber CSA in rotator cuff muscle after tendon tears. Collectively, while HIF-1α has been previously shown to promote fibrogenesis, the present data established that HIF-1α exerted divergent, cell type–specific effects within the injury microenvironment, functioning as a critical regulator of FAP cell differentiation fate by directing these progenitors toward adipogenesis rather than fibrogenesis. Interestingly, muscle CSA in FAP cell–specific HIF-1α knockout mice was found to be reduced. This may be because HIF-1α serves as a master transcriptional regulator of multiple trophic factors secreted by FAP cells, including vascular endothelial growth factor and IGF-1 (insulin-like growth factor 1), which are essential for myofiber survival, SC function, and muscle regeneration.^26,43^ The genetic ablation of HIF-1α in FAP cells would therefore disrupt this paracrine support network, leading to impaired myofiber maintenance even in the absence of direct myofiber-intrinsic defects. While our study focused on the chronic phase of muscle abnormalities to capture established fatty infiltration and fibrosis, longitudinal time course studies examining FAP cell dynamics and HIF-1α expression at earlier time points (1-2 weeks after injury) should be considered in the future and would provide valuable insights into the initial molecular events that drive FAP cell fate determination and subsequent pathological remodeling.

To further elucidate the effect of HIF-1α on the adipogenesis and fibrogenesis of FAP cells, we conducted additional experiments to examine the role of HIF-1α in regulating the differentiation of cultured FAP cells under normoxic conditions. CRISPR-Cas9–mediated HIF-1α knockdown in cultured FAP cells significantly reduced the expression of UCP1, a marker of brown adipose tissue (BAT) and increased the expression of α-SMA, a marker for fibrogenesis. Conversely, MK-8617–mediated PHD inhibition was accompanied by the increased expression of thermogenic adipogenic markers and the concurrent downregulation of fibrogenic markers in FAP cells, in association with the stabilization of HIF-1α proteins. It should be noted that MK-8617, as a PHD inhibitor, may exert pleiotropic effects beyond HIF-1α stabilization. The current study does not formally establish a causal role for HIF-1α in mediating the observed adipogenic fate shift in FAP cells. The correlative association between MK-8617 treatment and increased UCP1/PRDM16 expression may reflect HIF-1α–dependent, HIF-1α–independent, or combined mechanisms. The fact that the bidirectional manipulation of HIF-1α consistently shifted FAP cell differentiation fate under normoxic conditions provides compelling evidence that HIF-1α regulates the adipogenic-fibrogenic commitment of FAP cells through oxygen-independent mechanisms, likely downstream of injury-related paracrine signals rather than through classic hypoxia-driven HIF-1α stabilization.

BAT and beige fat defend body temperature homeostasis and regulate energy balance.^5^ UCP1 serves as a hallmark of brown/beige adipocytes. Besides its metabolic role, brown/beige fat can secrete several growth factors (eg, IGF-1 and follistatin) that promote muscle growth.^40^ A previous study revealed the significant contribution of BAT in promoting muscle regeneration after an RCT.^42^ Furthermore, the transplantation of beige FAP cells significantly reduced muscle degeneration and improved shoulder function after massive RCTs.^19^ The current study demonstrated that elevated HIF-1α expression induced the expression of UCP1 and other BAT markers in FAP cells. These findings suggest that HIF-1α may prompt FAP cells to differentiate into a promyogenic BAT phenotype. Concurrently, our in vivo data from FAP cell–specific HIF-1α knockout mice showed a reduction in myofiber size in SS muscles after RCTs compared to Cre^–^ controls. These data suggest that the loss of HIF-1α in FAP cells has an indirect effect on myocytes, probably through the promyogenic paracrine function of BAT-differentiated FAP cells. Future studies are needed to fully define the role of HIF-1α in regulating the brown adipogenic differentiation and paracrine function of FAPs. Overall, reduced HIF-1α levels in FAP cells during the later stage of an RCT are detrimental to FAP cell differentiation toward a favorable phenotype; thus, muscle exhibits severe fatty infiltration and muscle atrophy.

## Conclusion

This study sheds light on the cellular mechanisms underlying rotator cuff muscle degeneration after tendon tears. Despite a reduction in vascularity, the paradoxical decrease in global HIF-1α expression likely reflects denervation-induced reductions in metabolic demand. Muscle stem cells exhibited increased activation and proliferation after TTDN without significant changes in HIF-1α expression, and SC-specific HIF-1α deletion did not produce significant phenotypic consequences. HIF-1α emerges as a key player in FAP cell differentiation, promoting BAT differentiation while inhibiting fibrogenesis. Furthermore, HIF-1α may potentially influence the promyogenic paracrine function of FAP cells after RCTs. This novel insight holds promise for developing new treatment approaches targeting rotator cuff muscle atrophy, fibrosis, and fatty infiltration after tendon tears.

## Supplemental Material

sj-docx-1-ajs-10.1177_03635465261469689 – Supplemental material for Role of Hypoxia-Inducible Factor–1α in Regulating Muscle Degeneration After Rotator Cuff Tears Supplemental material, sj-docx-1-ajs-10.1177_03635465261469689 for Role of Hypoxia-Inducible Factor–1α in Regulating Muscle Degeneration After Rotator Cuff Tears by He Zhang, Austin Lee, Mengyao Liu, Agustin Diaz, Yizhao Zhang, Hubert T. Kim, Brian T. Feeley and Xuhui Liu in The American Journal of Sports Medicine
