# Supplementary material for: Role of Hypoxia-Inducible Factor–1α in Regulating Muscle Degeneration After Rotator Cuff Tears
Source: Am J Sports Med. 2026 Aug 12;54(11):2753–65. doi: 10.1177/03635465261469689 (PMC13530353; doi:10.1177/03635465261469689)
Supplement: Supplementary file 1 [file sj-docx-1-ajs-10.1177_03635465261469689.docx]

Supplementary Figure 1. HIF-1α mRNA expression in FAPs treated with PHD inhibitor compared to DMSO-treated controls. 18s was used as housekeeping gene. * *P*< 0.05 by Welch's t test.
